# Application of Photo-Fenton-Membrane Technology in Wastewater Treatment: A Review

**DOI:** 10.3390/membranes13040369

**Published:** 2023-03-23

**Authors:** Lihua Liang, Lin Ji, Zhaoyan Ma, Yuanyuan Ren, Shuyu Zhou, Xinchang Long, Chenyang Cao

**Affiliations:** 1 College of Urban and Environmental Science, Northwest University, Xi’an 710127, China; 2 Shaanxi Key Laboratory of Earth Surface System and Environmental Carrying Capacity, Xi’an 710127, China

**Keywords:** photo-Fenton, membrane, photocatalysis, wastewater treatment

## Abstract

Photo-Fenton coupled with membrane (photo-Fenton-membrane) technology offers great potential benefits in future wastewater treatment because it can not only degrade refractory organics, but also separate different pollutants from water; additionally, it often has a membrane-self-cleaning ability. In this review, three key factors of photo-Fenton-membrane technology, photo-Fenton catalysts, membrane materials and reactor configuration, are presented. Fe-based photo-Fenton catalysts include zero-valent iron, iron oxides, Fe-metal oxides composites and Fe-based metal–organic frameworks. Non-Fe-based photo-Fenton catalysts are related to other metallic compounds and carbon-based materials. Polymeric and ceramic membranes used in photo-Fenton-membrane technology are discussed. Additionally, two kinds of reactor configurations, immobilized reactor and suspension reactor, are introduced. Moreover, we summarize the applications of photo-Fenton-membrane technology in wastewater, such as separation and degradation of pollutants, removal of Cr(VI) and disinfection. In the last section, the future prospects of photo-Fenton-membrane technology are discussed.

## 1. Introduction

As a typical filtration technology, membrane technology has been widely researched and applied in wastewater treatment. It possesses several advantages such as high efficiency, small equipment size, easy operation, low energy consumption and low capital cost [1]. However, membrane technology can only separate chemicals, not break down pollutants, and even membrane bioreactors (MBRs) loaded with microorganisms are helpless for refractory organics. Meanwhile, the membrane-contamination phenomenon limits the effectiveness of practical applications due to the hydrophobic nature of membrane materials [2]. The accumulation of foulants on the membrane surface can block membrane pores resulting in a low flux of membrane, reduction in the membrane life and, finally, an increase in the cost of membrane technology [3]. Therefore, numerous researchers have considered combining membrane technology with advanced oxidation processes (AOPs) that can simultaneously break down resistant organic matter, separate different pollutants and make the membrane self-cleaning.

The Fenton reaction is one of the most studied AOPs. The Fenton reaction can be classified as a homogeneous and heterogeneous process. The classical Fenton reaction based on the interaction between ferrous iron (Fe^2+^) and hydrogen peroxide (H_2_O_2_) is defined as a homogeneous Fenton process, which is capable of degrading recalcitrant organic contaminants [4]. However, the homogeneous Fenton process has three main challenges: (1) the narrow optimal operating pH range of 2.8–3.5, (2) the generation of large amounts of iron sludge and (3) the difficulty in catalyst recovery [5]. Therefore, various types of heterogeneous catalysts (mainly solid iron-containing materials) have been extensively studied for heterogeneous Fenton reactions over the past few decades.

According to Wang and Tang [6], the heterogeneous Fenton reaction begins with the adsorption of the H_2_O_2_ molecule on surface catalysts. In this process, H_2_O_2_ is firstly adsorbed on the surface of the catalysts, and then bonded to surface-iron species (≡Fe^2+^ or ≡Fe^3+^) to form a surface complex (≡Fe^2+^ − H_2_O_2_ or ≡Fe^3+^ − H_2_O_2_) (Equations (1) and (2)). The ≡Fe^2+^ − H_2_O_2_ undergoes an intramolecular electron transfer to produce surface-bound hydroxyl radicals (•OH_surf_) with strong oxidation capacity to degrade organic pollutants in water (Equation (3)). The ≡Fe^2+^ produced in this process limits the Fenton reaction by consuming H_2_O_2_ and produces surface-bound hydroxyl peroxide radicals (•OOH_surf_) with weak oxidation capacity (Equation (4)). The •OOH_surf_ can further cause ≡Fe^2+^ regeneration (Equation (5)). The authors indicate that competing reactions occurring at the same time also consume reactive free radicals, leading to waste of H_2_O_2_ and reduced process efficiency.

(1) $$\equiv Fe^{2+}+H_{2}O_{2}\to \equiv Fe^{2+}-H_{2}O_{2}$$

(2) $$\equiv Fe^{3+}+H_{2}O_{2}\to \equiv Fe^{3+}-H_{2}O_{2}$$

(3) $$\equiv Fe^{2+}-H_{2}O_{2}\to \equiv Fe^{3+}+{\cdot OH}_{surf}+OH^{-}$$

(4) $$\equiv Fe^{3+}-H_{2}O_{2}\to \equiv Fe^{2+}+{\cdot OOH}_{surf}+H^{+}$$

(5) $$\equiv Fe^{3+}+{\cdot OOH}_{surf}\to \equiv Fe^{2+}+O_{2}+H^{+}$$

In general, some iron species inevitably leach from the Fe-based catalysts during the heterogeneous Fenton process, especially at low pH. Such dissolved iron ions can also initiate the homogeneous Fenton process (Equations (6) and (7)), which contributes to the further removal of organic compounds from the solution. However, the contribution ratio of heterogeneous and homogeneous Fenton to the overall degradation effect has not been determined.

(6) $$Fe^{2+}+H_{2}O_{2}\to Fe^{3+}+\cdot OH+OH^{-}$$

(7) $$Fe^{3+}+H_{2}O_{2}\to Fe^{2+}+\cdot OOH+H^{+}$$

At pH below 2.5, The formation of the [Fe(H_2_O)]^2+^ complex causes the substance to react slowly with H_2_O_2_, resulting in the low production of •OH, which reduces the efficiency of the Fenton process. On the contrary, the species [Fe(H_2_O)_8_(OH)_2_]^4+^, [Fe_2_(H_2_O)_7_(OH)_3_]^3+^ and [Fe_2_(H_2_O)_7_(OH)_4_]^5+^ are generated between pH 3 and 7, favoring the precipitation of iron and also resulting in lower efficiency of the process [7]. The homogeneous Fenton process produces a large amount of iron sludge, and sludge yield is substantially dependent upon the proportion and volume of the reagents [8]. To solve this problem, appropriate amounts of chelating agents (e.g., EDDS, EDTA, NTA) can be added to the Fenton systems. Chelating agents play an important role in both homogeneous and heterogeneous Fenton processes [9]. They can react with iron ions to form complexes, increase the solubility of iron ions and reduce precipitation [10]. Thus, the utilization rate of iron ions and the yield of active groups during the reaction can be improved. For Fe-based catalysts, due to the low leaching rate of iron ions, they can not only maintain a stable state at different pH, but also reduce the production of iron sludge in the application [11]. In addition, a previous study reported that when the ligands of chelating agents are adsorbed on mineral surfaces, they weaken the metal–oxygen bond, making it easier for metal ions to be released into the solution [12]. In addition to chelating agents, there is a similar strong complexation between iron ions and hydrophilic polymer molecules [13], which grants the membranes modified by hydrophilic polymer molecules a good photo-Fenton self-cleaning performance.

Recent studies have shown that the removal of pollutants from wastewater using the photo-Fenton reaction appears to be a highly promising approach. The photo-Fenton reaction overcomes the disadvantages of the conventional Fenton reaction, such as low pH suitability, generation of high iron sludge and difficulty in recovery [14]. In the photo-Fenton reaction, H_2_O_2_ undergoes photolysis to produce •OH (Equation (8)). The photo-Fenton reaction can also effectively alleviate the limitation of Fe^3+^ for the conventional Fenton reaction, where the irradiation of photon energy plays a role in the reduction of Fe^3+^ and the generation of the corresponding •OH (Equations (9) and (10)). [15,16,17]. Meanwhile, the organic intermediate can form a stable complex [Fe^3+^L] with Fe^3+^, which can absorb UV and visible light more efficiently and undergo photoreduction through a ligand-to-metal charge transfer (LMCT), generating Fe^2+^ (Equation (11)) [5].

(8) $$H_{2}O_{2}+hv\to \cdot OH$$

(9) $$\equiv Fe^{2+}+H_{2}O_{2}\to \equiv F{e(OH)}^{2+}+\cdot OH$$

(10) $$\equiv F{e(OH)}^{2+}+hv\to Fe^{2+}+\cdot OH$$

(11) $$\left[Fe^{3+}L\right]+hv\to {\left[Fe^{3+}L\right]}^{*}\to Fe^{2+}+L^{*}$$

Considering that the commonly used catalysts is semiconductor (e.g., Fe_2_O_3_, TiO_2_, g-C_3_N_4_), a photocatalytic reaction also occurs in the photo-Fenton process. The reduction of Fe^3+^ to Fe^2+^ by the action of photogenerated electrons and H_2_O_2_ regenerates Fe^2+^ (Equations (12)–(18)), promotes the cycle between Fe^2+^/Fe^3+^ and produces more •OH, which enhances the oxidation capacity of the system.

(12) $$Catalysts+hv\to h^{+}+e^{-}$$

(13) $$h^{+}+H_{2}O\to \cdot OH+H^{+}$$

(14) $$h^{+}+OH^{-}\to \cdot OH$$

(15) $$e^{-}{+O}_{2}\to \cdot O_{2}^{-}$$

(16) $$\cdot O_{2}^{-}+2H^{+}+e^{-}\to H_{2}O_{2}$$

(17) $$H_{2}O_{2}+e^{-}\to \cdot OH+OH^{-}$$

(18) $$Fe^{3+}+e^{-}\to Fe^{2+}$$

The •OH generated by the photo-Fenton reaction and h^+^ produced by the photocatalytic process oxidize with organic pollutants in wastewater and eventually mineralize and decompose the pollutants into CO_2_ and H_2_O (Equation (19)).

(19) $$\cdot OH/h^{+}+organics\to H_{2}O+degradation\;products\to H_{2}O+CO_{2}$$

Photo-Fenton-membrane technology allows the system to perform both filtration and degradation functions. In the filtration process, contaminants are trapped on the membrane surface and pores. The contaminants are then degraded by free radicals generated in situ by catalyst-activated hydrogen peroxide, driven by UV or visible light [18,19].

This review firstly introduces several photo-Fenton catalysts and membrane materials which have been applied in wastewater treatment by photo-Fenton-membrane technology. Then, the currently available photo-Fenton-membrane reactors are introduced. Finally, the main applications of photo-Fenton-membrane technology are summarized. This review analyzes the research basis and frontier of photo-Fenton-membrane technology and related reactors, and discusses the feasibility of photo-Fenton-reaction-coupling-membrane technology. The problems to be solved in the current research process are pointed out, which provides a theoretical basis and further research directions for realizing the application of this technology in engineering.

## 2. Photo-Fenton Catalysts and Membranes

### 2.1. Photo-Fenton Catalysts

#### 2.1.1. Fe-Based Materials

In the Fenton reaction, the rate of reaction between Fe^2+^ and H_2_O_2_ to form •OH is faster than that of Fe^3+^ to form HO_2_•, and the reactivity of •OH is higher than that of HO_2_• [20]. High Fe^3+^ content can limit the overall efficiency of the Fenton reaction. Therefore, how to accelerate the reduction of Fe^3+^ to Fe^2+^ is key to improving the efficiency of the reaction [11]. In the photo-Fenton system with Fe-based materials as catalysts, the reduction of Fe^3+^ is accelerated and the production of •OH is promoted, which accelerates the degradation rate of the system for organic matter. It can also overcome the disadvantages of the traditional Fenton method such as difficult separation of catalysts, narrow pH applicability range, high yield of iron sludge and causing secondary pollution.

Common Fe-based materials include zero-valent iron (ZVI), iron oxides, Fe-based multiple oxides and metal–organic frameworks (MOFs).

##### ZVI

Among the Fe-based catalysts, ZVI has the lowest valence state and is highly reductive. It can react with either H_2_O_2_ or O_2_ to form Fe^2+^, or with Fe^3+^ to form Fe^2+^ [21]. Although the ZVI-based Fenton reaction requires acidic conditions to be truly successful in removing contaminants, the addition of iron chelators or the use of a separation method (the first step uses ZVI alone, followed by a second step of the photo-Fenton process) can also enhance their contaminant degradation rates at a neutral pH [22]. Thus, ZVI can be applied to the study of efficient degradation of organic pollutants by photo-Fenton. A significant improvement in the efficiency of the ZVI-catalyzed Fenton reaction under light has been reported [23]. Due to the nature of metal compounds, ZVI with high interfacial energy loaded on the membrane surface can also enhance the hydrophilicity of the membrane surface and improve the antifouling performance of the membrane. However, there are few studies on the combination of ZVI with membrane treatment for the photo-Fenton reaction [24]. Lee et al. [24] prepared a photo-Fenton-active membrane via in situ functionalization of polypyrrole (PPy) and ZVI nanoparticles and loaded it on polyvinylidene fluoride (PVDF). The membrane has high surface wettability, photothermal properties and photo-Fenton reactivity. Under the condition of H_2_O_2_ addition of 10 mmol·L^−1^ and initial concentration of MB of 10 mg/L, the degradation rate reached 99% after 90 min of reaction.

##### Iron Oxides

In nature, there exist many kinds of iron–oxygen minerals, which contain iron oxides, such as hematite (α-Fe_2_O_3_), magnetite (Fe_3_O_4_) and goethite (β-FeOOH). These iron oxides have some reactivity under the photo-Fenton reaction. Iron oxides can be applied as catalysts in the study of pollutant degradation in wastewater by the photo-Fenton reaction because of their low production cost, simple method and low toxicity. Feng et al. [25] prepared a low-crystalline Fe_2_O_3_@carboxylic multiwalled carbon-nanotube ceramic membrane and evaluated its degradation ability as an aromatic compound. After 120 min of photo-Fenton reaction and continuous filtration, the membrane achieved more than 90% removal of phenol and atrazine with good self-cleaning performance. Yan et al. [26] loaded α-Fe_2_O_3_ prepared by the solvothermal method onto a flat ceramic membrane (CM) by spray printing and low-temperature sintering to form an α-Fe_2_O_3_-CM photo-Fenton reactive membrane with good separation performance and reusability. The degradation rate of tetracycline hydrochloride reached 82% after 80 min of photo-Fenton reaction. Xie et al. [27] assembled green tannic acid (TA)-Fe(III) complexes onto the surface of PVDF, followed by in situ mineralization of β-FeOOH, to prepare PVDF/TA/β-FeOOH membranes for effective oil-/water-emulsion separation. The prepared membranes showed fast and excellent flux recovery, due to the powerful photo-Fenton catalytic activity of β-FeOOH.

##### Fe–Metal-Oxide Composites

The metal oxides of Bi, Ni and Cu are sensitive to light in the ultraviolet-visible wavelength. If these oxides are doped with Fe, the degradation rate of the Fenton reaction can be increased and the degradation efficiency of the photo-Fenton reaction can be enhanced [28]. Gao et al. [29] prepared a NiFe_2_O_4_/TA/PVDF membrane by an in situ deposition method. The membrane has good superhydrophilic and underwater-superoleophobic properties and can effectively separate emulsified oils from representative oil-in-water emulsions with a separation efficiency above 99%. The membrane also exhibits good treatment efficiency, antifouling performance and good reusability when treating mixed organic contaminants (water-insoluble emulsified oil and water-soluble organic dyes) present in the same water system.

##### Fe-Based MOFs

MOFs are a new type of inorganic/organic filler composed of metal ions and organic ligands for the construction of high-performance membranes. Recently, MOFs have attracted considerable interest, as they showed potential for concurrent heterogeneous Fenton-like oxidation [30] and photocatalysis processes [31,32]. It has been shown that Fe-based MOFs can absorb visible light and perform photocatalytic processes for water purification [33,34,35]. Yue et al. [36] prepared a graphene oxide (GO)/Ag/M88A membrane by using a partially reduced photocatalyst on a PVDF substrate membrane. The membrane showed good performance of separation (99.7%) and degradation (90% degradation in 60 min) of MB.

#### 2.1.2. Non-Fe-Based Materials

Apart from Fe-based materials, some transition-metal materials have shown good performance in photo-Fenton systems, such as TiO_2_ [37], CuO [38], ZrO_2_ [39] and Co_3_O_4_ [40]. Mokoba et al. [41] prepared a BiOBr@Co_3_O_4_ nanostructure composite membrane on copper mesh substrates by hydrothermal synthesis and calcination of Co_3_O_4_ nanowire clusters, and hydrothermal deposition of BiOBr photocatalysts. The membrane has superhydrophilic and underwater-superoleophobic properties. The emulsion separation efficiency was 99.98% and 98.66% for surfactant-free and surfactant-stabilized oil-in-water emulsions, respectively.

In recent years, nonmetallic compounds represented by C_3_N_4_ have also received attention as photo-Fenton catalysts. Compared to metal-compound catalysts, C_3_N_4_ is more chemically stable and suitable for use in neutral pH environments. Lan et al. [42] used a self-assembly technique to prepare a graphitic carbon nitride(g-C_3_N_4_)/Fe-containing-polyoxometalate (Fe-POM) photo-Fenton membrane with good filtration and catalytic-degradation properties. The MB was basically degraded completely after 80 min of reaction. Yue et al. [43] prepared porous potassium-doped g-C_3_N_4_ membranes coated with polyvinylpyrrolidone (PVP) by the vacuum-filtration method. The prepared membranes exhibited enhanced hydrophilicity, underwater superoleophobicity and high photo-Fenton activity.

### 2.2. Membranes

The main materials used in membrane technology are polymers and ceramics [44]. Many polymers are used as membrane substrates for photo-Fenton catalysts due to their good thermal stability, ease of synthesis and low cost, such as PVDF [3,29,45], PVP [43], polysulfone (PSF) [46] and polydopamine (PDA) [47]. The photocatalytic activity of the different polymer membranes was mainly related to their ability to release soluble iron. González-Bahamón et al. [48] used the photo-Fenton method to immobilize iron on four different polymers (polyethylene (PE), polypropylene (PP), high-impact polystyrene (PS) and polymethylmethacrylate (PA)) and performed a comparative test of the degradation efficiency of m-diphenol. The results showed that the best photocatalytic activity was achieved with PE and PP membranes as carriers, and that the higher resorcinol conversion and mineralization rates were related to iron dissolution.

However, the polymeric membrane is not resistant to UV irradiation and free-radical damage, resulting in its short service life in practice. This is discussed in detail in Section 4.4.1. Ceramic membranes made of alumina, zirconia or titanium dioxide exhibit higher porosity, chemical stability, mechanical strength and longer service life than polymeric membranes [49]. Dennis et al. [39,50] prepared the Ce-Y-ZrO_2_/TiO_2_ photocatalytic ultrafiltration membrane by the sol–gel method. The membrane was used for filtration and photo-Fenton treatment of municipal-wastewater-treatment-plant (UWWTP) wastewater at neutral pH. The membrane significantly reduced the turbidity of the UWWTP effluent and significantly improved the degradation efficiency of the subsequent solar-photo-Fenton treatment.

In addition, some researchers coat the membrane surface with hydrophilic substances to improve the antifouling properties of the membrane. For example, chitosan (CS) is hydrophilic. CS coating enhances the surface hydrophilicity of the membrane. The increase in membrane hydrophilicity helps improve the antifouling properties of the membrane [51]. Zheng et al. [52] successfully prepared the highly hydrophilic CS/polyacrylonitrile (PAN)@FeOOH/g-C_3_N_4_ membrane with self-cleaning ability by coating the prepared PAN@FeOOH/g-C_3_N_4_ nanofibers with purified CS. The results showed that the water-contact angle was reduced to less than 40° after coating the membrane with CS.

## 3. Photo-Fenton-Membrane Reactors

The application of the photo-Fenton-membrane technology requires catalysts, membrane modules, light sources and other components to build a photo-Fenton-membrane reactor. The photo-Fenton-membrane reactors can be divided into immobilized reactors and suspension reactors according to the state of the catalysts.

### 3.1. Immobilized Reactors

**Figure 1:**
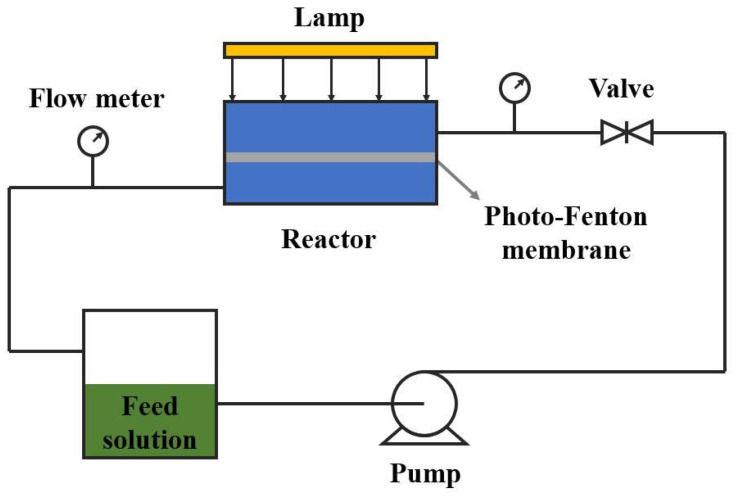
Schematic diagram of common immobilized reactors.

Figure 1 shows the schematic diagram of common immobilized reactors. Immobilized reactors can be divided into three types according to the way the catalyst is combined with the membrane: (1) membranes made by mixing with catalysts, (2) membranes made by fixing catalysts on the surface and (3) membranes made independently of catalysts.

#### 3.1.1. Membranes Made by Mixing with Catalysts

This type combines catalyst particles with a polymer matrix through a co-blending method. The photo-Fenton reaction is used to efficiently degrade the contaminants attached to the membrane surface. Wang et al. [53] prepared an Fe-complex-loaded visible-light-active cellulose-acetate (CA)-composite membrane (Fe-complex/CA) by the band-casting method. Under the condition of low concentration of H_2_O_2_, the Fe-complex/CA-composite membrane showed good visible photocatalytic activity against organic dyes and sulfadiazine antibiotics. The degradation rates of basic magenta, MB and sulfadiazine reached 100%, 93.4% and 95.7%, respectively, after 60 min of photo-Fenton reaction. Liu et al. [54] prepared a ZIF-67-embedded PVDF (ZIF-67@PVDF) hybrid matrix ultrafiltration membrane using nonsolvent-induced phase-separation technology for the efficient degradation of dye wastewater. The peroxymonosulfate-online-cleaning-assisted ZIF-67@PVDF membrane achieved high dye removal efficiency for orange II (AO7, 97.3%), MB (98.2%), RhB (90.5%) at a high pH of 11.

Although the co-blending method is simple and efficient, the addition of photocatalysts in the co-blended membrane system is usually required to be no more than 1%, considering the mechanical strength and membrane performance. Additionally, most of the catalysts are embedded inside the membrane rather than on the surface, resulting in low degradation efficiency [55]. In addition, the inorganic nanomaterials are poorly compatible with the soft-polymer matrix, which can lead to poor aggregation and dispersion of nanoparticles or nonselective interfacial voids, weakening the separation performance of the membrane [56].

#### 3.1.2. Membranes Made by Fixing Catalysts on the Surface

This type is made by loading catalyst particles onto the membrane surface by surface coating, surface grafting, etc., or depositing or coating a catalytically active membrane on the surface of the membrane module to mitigate the membrane-contamination phenomenon by using the photo-Fenton reaction to efficiently degrade the pollutants attached to the membrane surface.

Chen et al. [13] obtained the (polyvinylidene fluoride)-g-(polyacrylic acid)(PVDF-g-PAA) ultrafiltration membrane with visible-light photo-Fenton self-cleaning performance by mineralizing Fe^3+^ into β-FeOOH nanoparticles deposited on the membrane surface through the strong complexation of Fe^3+^ with the abundant carboxyl groups on the ultrafiltration membrane surface. After 60 min of reaction, the degradation rate of MB by the ultrafiltration membrane was 95.7%. Additionally, it showed good self-cleaning and recycling performance.

Gao et al. [29] prepared a NiFe_2_O_4_/TA/PVDF composite membrane by the in situ deposition method under a vacuum system. On the one hand, the composite membrane exhibited an efficient photo-Fenton degradation rate for organic dyes. On the other hand, it can effectively separate emulsified oil and representative oil-in-water emulsions, both with separation efficiencies above 99% and high fluxes.

Zheng et al. [57] bonded nanoparticles with adhesion and superhydrophilicity to the surface of MIL-88A. The modified MIL-88A was then hydrophilically coated on the PVDF membrane surface by filtration to obtain a composite photo-Fenton antifouling membrane. The membrane maintained a flux close to 5000 L m^−2^·h^−1^·bar^−1^ in at least 10 separations. The dye removal by photo-Fenton reaction was maintained at more than 99.6%.

However, this method has certain disadvantages, such as the binding of catalyst particles to the membranes, which can affect the degradation efficiency.

#### 3.1.3. Membranes Made Independently of Catalysts

**Figure 2:**
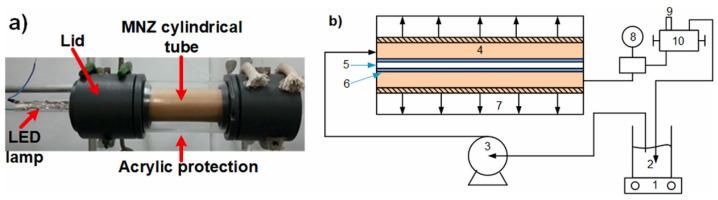
(**a**) Membrane module, (**b**) reaction system schematic diagram: (1) magnetic stirrer, (2) feed solution, (3) diaphragm pump, (4) MNZ cylindrical membrane, (5) lamp, (6) quartz tube, (7) acrylic tube, (8) pressure gauge, (9) sampling, (10) valve [58].

In addition to the above-mentioned immobilization methods, the catalyst can also be made directly into an independent photo-Fenton membrane without support. Domenzain-Gonzalez et al. [58] used an extrusion method to prepare mesoporous cylindrical membranes by mixing Mexican natural zeolite (MNZ) with additives to form a paste with a 67.8% solids content and drying it. Then, the cylindrical membrane was arranged as a photocatalytic membrane reactor for the degradation of RB5 dye. The oxidation states of MNZ are Fe^2+^ and Fe^3+^. The membrane module and reactor are shown in Figure 2. The difference between this reactor and the one in Figure 1 is that the lamp is placed in the cylindrical film, which improves the light-absorption efficiency.

Molina et al. [59] used the impregnated coating method to immobilize the needle-ferrite membrane on the walls of the photo-Fenton reactor and obtained homogeneous, stable and optimally active needle-ferrite layers at a needle-ferrite-suspension concentration of 15 g·L^−1^. A mixture of six selected drug compounds (nicotine, 4-acetylaminoantipyrine, hydrochlorothiazide, ranitidine, diclofenac sodium and sulfamethoxazole) was degraded in an aqueous solution. The 100% degradation of the selected compounds was carried out after 6 h of reaction, except for ranitidine and nicotine.

This method can effectively avoid the reduction in photo-Fenton-reaction activity caused by the overlapping and shedding of catalyst particles to improve the thermal, mechanical and chemical stability of the membrane.

### 3.2. Suspension Reactors

**Figure 3:**
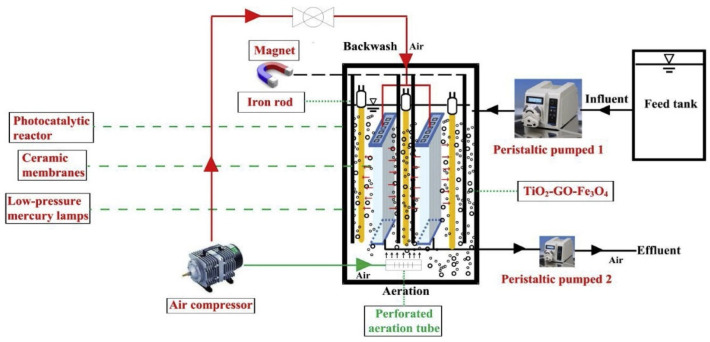
Schematic diagram of SMSMPR [60].

Considering that immobilized reactors reduce the specific surface area and reaction activity of catalysts, and some researchers have tried to construct suspension reactors. In this type of reactor, the catalyst particles are uniformly suspended in the aqueous solution, and their specific surface area and reactivity are substantially increased. Li et al. [60] designed an immersed magnetic-separation membrane-photocatalytic reactor (SMSMPR) and combined it with a TiO_2_-GO-Fe_3_O_4_ magnetic catalyst to achieve the recovery of catalysts using an applied magnetic field. The schematic diagram of SMSMPR is shown in Figure 3. The ceramic membranes used in SMSMPR provided backwash treatment, enhancing its self-purification capability.

Wang et al. [61] constructed a submerged membrane photocatalytic reactor (SMPR) with Fe(III)-ZnS/g-C_3_N_4_ as a photo-Fenton catalyst for the deep treatment of nitrobenzene-phenol (PNP) wastewater. When the influent PNP concentration was 10 mg·L^−1^, the initial pH was 5, the catalyst dosage was 1.0 g·L^−1^, the H_2_O_2_ concentration was 170 mg·L^−1^, the aeration rate was 0.50 m^3^·h^−1^ and the operation time was 4 h; the PNP removal rate was 91.6% by the SMPR under simulated solar-light irradiation. Moreover, the recovery rate of catalysts reached 100%.

## 4. Application of Photo-Fenton-Membrane Technology

Currently, photo-Fenton-membrane technology has obvious advantages in wastewater treatment, including the separation and degradation of pollutants, removal of harmful heavy metal ions and disinfection. In this section, the main applications of this technology are summarized.

### 4.1. Separation of Pollutants

In the field of wastewater treatment, especially oily wastewater treatment, membrane-separation technology has become the most dominant means. It can remove impurities or complete the separation of oil and water. In the process of oil–water separation, the surface of the separation membrane is easily contaminated by the organic matter in the wastewater, which affects the performance of the separation membrane. The photo-Fenton process can degrade contaminants by generating hydroxyl radicals, and its combination with membrane filtration may be an effective way to improve the antifouling properties and filtration performance of membranes [62]. Improving the hydrophilicity of the membrane and building a hydration layer to avoid the adsorption and deposition of contaminants on the membrane is also a common antifouling strategy [63]. Generally, high surface energy and surface roughness lead to membranes with superhydrophilic and underwater-superoleophobic properties [64,65,66]. The underwater-anti-oil-adhering characteristic of the membrane surface can prevent oils from adhering and blocking the channels during separating the oily sewage [67]. Additionally, it is possible to reduce the interaction force between contaminants and the membrane surface by constructing a membrane with low surface energy, so that the attached contaminants can be easily removed from the deposited membrane [68,69]. However, this reduces the permeability of the membrane [70,71].

As described in Table 1, photo-Fenton membranes exhibit versatile separation properties against different types of foulants. Among them, polymer-based membranes occupy the main market due to their low cost and simple fabrication methods. Water-contact angle (WCA) and underwater oil-contact angle (OCA) are commonly used to describe the hydrophilicity/hydrophobicity of a membrane. Hydrophilic membranes (WCA ≤ 90°) have higher flux (1500–7500 L·m^−2^·h^−1^·bar^−1^) and separation efficiency than other membranes and are widely used in membrane-modification studies. Fe-based MOFs (e.g., M88A, M88B) are often used as catalysts for photo-Fenton-membranes due to their good catalytic properties and improved membrane-wetting performance. After the introduction of micron or nanocatalyst particles on the membrane to increase the surface roughness, the membrane can obtain special wettability of superhydrophilic, superoleophilic, superhydrophobic and superoleophobic, thus having better oil–water separation properties. Additionally, superhydrophobic–superoleophobic membranes are mainly used for the separation of oil-in-water emulsions, and superhydrophilic–submerged-superoleophobic membranes are mainly used for the separation of water-in-oil emulsions [72]. Meanwhile, the addition of photo-Fenton makes membrane fouling degradable, thus reducing membrane contamination and achieving the self-cleaning of membranes. However, the introduction of catalyst particles may also change the pore size of the membrane. It is possible to adjust the pore size of the modified membrane to offer the “size-serving effect” and guarantee high separation efficiency for oily emulsified wastewater [67]. It may also make separation more difficult. For example, inorganic nanomaterials have bad compatibility with soft-polymeric matrices, which leads to aggregation and poor dispersion of nanoparticles or nonselective interfacial voids weakening the separation properties of membranes [56]. Overall, the photo-Fenton catalysts complicate the separation capacity of membranes.

In addition to the influence of membrane properties, a number of condition factors in the filtration process can also affect the separation performance of the membrane, such as the initial concentration of the pollutants and influent flux [73].

**Table 1:** Separation performance of different photo-Fenton membranes.

Membranes	WCA	OCA	Substances to Be Separated	Flux/(L·m^−2^·h^−1^·bar^−1^)	Separation Efficiency (%)	Ref.
GO/M88A	0–77.6°	126.6–161.7°	MB	28.7	99.58	[74]
			RhB	26.3	99.95	
			MO	30.3	98.9	
PVDF/NM88B	62.3–84.2°	108.9–151.3°	hexane	679	all above 99.4	[45]
			toluene	1766		
			petroleum ether	1970		
			soybean oil	340		
			dichloroethane	1359		
PVDF/PPy/ZVI	26.4°	139.2°	soybean oil	2672.7	97	[24]
CS/PAN@FeOOH/g-C_3_N_4_	40°	/	MB	130.5	68.49	[52]
			ERY	182.7	72.58	
GO-2	34.6	/	MLB	30	93.1	[75]
prGO/Ag/M88A	0–56.1°	/	EB	85.8	95.8	[36]
PS/HTCC-3/FcSO_3_	≈84°	/	BPS	1.4	98.1	[76]
PMIA/β-FeOOH	0°	>150°	various oil–water mixtures	>13,925	>97	[77]
SPAN@GO/M88A	17.8 ± 3.5°	159.5 ± 2.1°	oily wastewater	1569 ± 104	99.82	[67]
PVP-coated porous potassium-doped g-C_3_N_4_	8.0°	158.6°	toluene	94.1	99.3	[43]
			n-hexane	104.1	99.6	
			dichloromethane	100.8	99.5	
PVDF/TA-Fe (III)-HPO4	0°	155.50°	oil-in-water	1840.94	/	[78]
PVDF/TA/β-FeOOH	0°	156.4°	petroleum ether	1698.5	99.7	[27]
NiFe_2_O_4_/TA/PVDF	0°	159.1°	toluene	1477	>99.35	[29]
NM88B@QFM	0°	161.3°	petroleum ether	67,900	>99.4	[79]
PVDF-g-PAA@FeOOH	0°	/	bovine serum albumin	/	>90	[13]
PAA@NM88B/GO	21.8°	/	MB	~62.5	>97.5	[80]
PSF + β-FeOOH	~29°	/	CR	100	92.6	[62]
			RB	105.5	60.6	
			MB	53.6	86	
β-FeOOH/HNTs@PVDF	0°	>150°	surfactant-free n-hexane oil-in-water emulsion	7338 ± 222	>99	[81]
PVDF-MT	0°	151.38°	carbon tetrachloride	6133.86	/	[82]

### 4.2. Degradation of Pollutants

There have been many studies on the application of photo-Fenton-membrane technology in practical wastewater treatment, including flax wastewater [83], oil-refinery wastewater [84], winery wastewater [85], textile-industry wastewater [86] and paper-industry wastewater [87]. Several studies using photo-Fenton-membrane technology for contaminant degradation are listed in Table 2. At present, the degradation performance of photo-Fenton membranes is mainly studied for dyes, antibiotics and drugs. In addition to pH, the degradation process of pollutants by photo-Fenton membranes is influenced by the amount of H_2_O_2_, the initial concentration of pollutants and light. The ratio of Fe^2+^/H_2_O_2_ should be maintained at a certain level, which is determined by the nature of the wastewater [88]. According to the existing research results, the dosage of 5–50 mmol/L H_2_O_2_ is appropriate. In addition, the light sources used in the research on polymer membranes are usually in the visible light range to avoid UV damage to the polymer [52,53,77]. In contrast, studies on other membrane materials use more UV lamps or apply light sources with wavelengths in the UV range [76,89,90].

The selection of various catalysts needs to be further optimized when different membrane types and technologies are combined. At the same time, the use of UV lamps increases energy consumption substantially, and the consumption of H_2_O_2_ increases the unit operating cost of the system [91]. The operating cost of the system is also a major factor to be considered when putting it into practical application. Studies guiding the selection of the optimal photo-Fenton-reaction/membrane-separation process for various treatment applications of wastewater generated by important industries are still scarce.

**Table 2:** Degradation performance and experimental conditions of different photo-Fenton membranes.

Membranes	Pollutants	Dosage of H_2_O_2_/(mmol·L^−1^)	Initial Concentration of Pollutants/(mg/L)	Light Conditions	Time/min	Degradation Rate (%)	Ref.
GO/MIL-88A(Fe)	BPA	10	10	visible light (λ ≥ 420 nm)	40	97.27	[75]
	MB	10	10	visible light (λ ≥ 420 nm)	40	98.81	
Fe_2_O_3_@CMWCNTs	phenol	3	2.5	xenon lamp	120	93	[25]
α-FeOOH-coated ceramic membrane	bovine serum albumin	10	35	UV lamp (401 μW·cm^−2^)	60	86	[89]
	humic acid	10	25	UV lamp (401 μW·cm^−2^)	60	76.4	
CS/PAN@FeOOH/g-C_3_N_4_	MB	50	50	xenon lamp (λ > 400 nm)	80	100	[52]
	ERY	50	20	xenon lamp (λ > 400 nm)	140	100	
prGO/Ag/M88A	MB	35	10	visible light (300 W, λ ≥ 420 nm)	60	98.8	[36]
FeOCl-coated ceramic membrane	nitrobenzene	8	1.23	UV (λ = 254 nm)	7	100	[90]
(3D) CuO/TiO_2_	RhB	88	50	mercury–xenon arc lamp	10	100	[38]
PS/HTCC-3/FcSO_3_	BPS	1	20	UV lamp (6 W, 254 nm)	60	99.5	[76]
PMIA/β-FeOOH	MB	30 wt%, 20 μL	10	LED light (400~800 nm)	360	99.99	[77]
	RhB	30 wt%, 20 μL	10	LED light (400~800 nm)	480	99.75	
TiO_2_-GO-Fe_3_O_4_	amoxicillin	20	20	visible light	120	88.5	[92]
SPAN@GO/M88A	MB	30 wt%, 50 μL	50	xenon arc lamp (150 W)	20	>90	[67]
TiO_2_-ZrO_2_ UF	caffeine	1.5	0.1	xenon lamp (300~800 nm)	15	66	[39]
	imidacloprid	1.5	0.1	xenon lamp (300~800 nm)	15	52	
	thiacloprid	1.5	0.1	xenon lamp (300~800 nm)	15	45	
	carbamazepine	1.5	0.1	xenon lamp (300~800 nm)	15	83	
Fe(III)-ZnS/g-C_3_N_4_	p-nitrophenol	5	10	xenon lamp (500 W)	240	91.6	[61]
Fe-complex/CA	MB	39	10	xenon lamp (400~780 nm)	60	100	[53]
Co_3_O_4_@Fe_3_O_4_/cellulose	perfluorooctanoic acid	30	20	visible light	180	94.5	[93]
PVDF-MT	MB	10	10	xenon lamp	70	98	[57]
α-Fe_2_O_3_-CM	tetracycline hydrochloride	19.4	20	visible LED light	200	82	[26]

### 4.3. Additional Function

Fenton oxidation is useful for the complete removal of Cr(VI) [94], indicating the potential of the photo-Fenton-membrane technology in the removal of Cr(VI) from wastewater. Under acidic conditions, Fe^2+^ can reduce Cr(VI) to Cr(III) (Equation (20)). Piao et al. [77] used the prepared PMIA/β-FeOOH nanofiber membrane to reduce the highly toxic Cr(VI) to Cr(III) in water. After 6 h of reaction, hexavalent chromium was almost completely removed.

(20) $${Cr}_{2}O_{7}^{2-}+6{Fe}^{2+}+14H^{+}\to 2{Cr}^{3+}+6{Fe}^{3+}+7H_{2}O$$

The photo-Fenton reaction is also a common means of disinfection [95]. Rincon and Pulgarin performed the first practical photo-Fenton reaction for microbial inactivation at near-neutral pH [96]. In the last decade or so, much work has been conducted on the photo-Fenton reaction used for disinfection, including the disinfection effect of different catalysts [97,98], the disinfection effect on different bacteria [99,100], the factors affecting the photo-Fenton-disinfection effect [101,102] and the mechanism of disinfection [103,104]. The exact mechanism of sterilization at neutral pH has not yet been determined. It is widely believed that solar irradiation leads to a large accumulation of reactive oxygen species in the cell, causing bacterial death. The addition of H_2_O_2_ further disrupts the balance of internal reactive oxygen species (ROS). The addition of Fe also induces a ligand–metal-charge transfer-mediated-inactivation mechanism due to cell-wall damage and external ROS [105]. Xue et al. [106] prepared an N-Fe (III)@PCN-222/PDA/PVDF composite membrane with powerful photocatalytic bactericidal performance. The production of •O_2_^−^, •OH and ^1^O_2_ during the reaction played a key role in bacterial inactivation.

It is worth mentioning that in addition to considering the photo-Fenton reaction and membrane technology as a whole, some studies have also used the photo-Fenton process as a pretreatment or post-treatment process for membrane reactors. The pretreatment process can decompose the hard-to-degrade substances, improve the biodegradability of the biological treatment stage and reduce the biological toxicity into the bioreactor. Ballesteros Martin et al. [107] used a coupled AOP-MBR system to treat wastewater containing high concentrations of pesticides. A photo-Fenton pretreatment (34% mineralization) was carried out, and the MBR treatment resulted in a biodegradation rate of 98%. Additionally, the activated sludge population did not change much during the biological treatment process and still maintained acceptable biodegradation efficiency. As a post-treatment process, the photo-Fenton reaction has lower chemical costs and less iron sludge emissions, and can remove more degradable contaminants. Ito Sakurai et al. [108] used the photo-Fenton process as a post-treatment process complementary to ultrafiltration and microfiltration membranes, and the results showed that the photo-Fenton reaction could significantly improve the COD and color removal of wastewater.

### 4.4. Problems

Although photo-Fenton-membrane technology has shown remarkable potential in many fields of application, there are still some problems.

#### 4.4.1. The Service Life of Membrane

As the most widely used membrane material, polymer membranes may deteriorate when exposed to UV light directly during treatment. UV radiation causes photo-oxidative degradation, which results in the breaking of the polymer chains, produces free radicals and reduces the molecular weight, causing the deterioration of mechanical properties [109]. Lee et al. [110] found that cracks and fractures were observed in the outer surface layer of the PVDF-TiO_2_ composite membrane when the membrane was exposed to 120 h of UV light. The permeation flux of the irradiated membrane increased from 10.89 L/m^2^·h to 21.84 L/m^2^·h, while the oil-retention rate decreased with increasing UV-exposure time.

In addition, the immobilized catalysts may also absorb UV-light energy, leading to membrane aging, and further change its surface morphology and treatment performance. To avoid this damage, instead of using a UV light source, a visible light source can be used, as indicated in Table 2.

#### 4.4.2. Small Treatment Scale

At present, the application of photo-Fenton-membrane technology is mainly focused on laboratory experiments or small-scale trials. If this technology is to be scaled up to large-scale applications, special attention needs to be paid to the following aspects. First is the energy consumption and cost issues. On the one hand, the photo-Fenton process requires many chemicals, including acidifiers and H_2_O_2_. On the other hand, the widespread use of lamps dramatically increases energy consumption and costs. At the same time, in large reactors, it is often difficult for the light to penetrate the effluent and activate the catalysts deep in the water due to the interference of suspended substances in the effluent and the limitation of the reactor structure, resulting in low overall treatment efficiency.

## 5. Conclusions

This review summarizes the research progress of photo-Fenton-membrane technology in wastewater treatment. Several representative types of photo-Fenton catalysts and membrane materials (ZVI, iron oxides, Fe-based multiple oxides and MOFs), as well as several typical reactor models, are highlighted. Photo-Fenton-membrane technology has been studied and applied more in the field of oil–water separation and pollutant removal, but few in the removal of heavy metals from wastewater and sterilization and disinfection. Past studies on photo-Fenton technology in these areas have shown that photo-Fenton-membrane technology still has great potential which remains to be exploited and should receive more attention. Although photo-Fenton-membrane technology integrates the inherent advantages of photocatalysis, Fenton oxidation and membrane treatment, and it has shown excellent results in water purification, it still faces many challenges and difficulties:

- The life of the membrane is unstable. The membrane is susceptible to damage to the membrane material and structure when exposed to light. Moreover, after the membrane is recycled several times, the loaded catalyst particles tend to fall off from the membrane surface, affecting the subsequent treatment effect and making it difficult to recover.
- Catalyst selection should be cautious. Careful selection of the appropriate photo-Fenton catalyst is required because when the catalysts are combined with the membranes through co-blending or loading, they can affect the hydrophilicity/hydrophobicity of the membranes and have an impact on the separation effect.
- The optimization of the process needs to be improved. The optimization of the photo-Fenton-membrane treatment process can be carried out through strategies such as objective function development and selection of decision variables.

When all the above is addressed, photo-Fenton-membrane technology can play a bigger role in wastewater treatment.

## Data Availability

Not applicable.

## References

[1] Obotey Ezugbe E., Rathilal S. (2020). Membrane Technologies in Wastewater Treatment: A Review. Membranes.

[2] Teng J., Wu M., Chen J., Lin H., He Y. (2020). Different fouling propensities of loosely and tightly bound extracellular polymeric substances (EPSs) and the related fouling mechanisms in a membrane bioreactor. Chemosphere.

[3] Wang T., Wang Z., Wang P., Tang Y. (2019). An integration of photo-Fenton and membrane process for water treatment by a PVDF@CuFe_2_O_4_ catalytic membrane. J. Membr. Sci..

[4] Wang J., Wang S. (2020). Reactive species in advanced oxidation processes: Formation, identification and reaction mechanism. Chem. Eng. J..

[5] Clarizia L., Russo D., Di Somma I., Marotta R., Andreozzi R. (2017). Homogeneous photo-Fenton processes at near neutral pH: A review. Appl. Catal. B Environ..

[6] Wang J., Tang J. (2021). Fe-based Fenton-like catalysts for water treatment: Catalytic mechanisms and applications. J. Mol. Liq..

[7] Sanabria P., Wilde M.L., Ruiz-Padillo A., Sirtori C. (2022). Trends in Fenton and photo-Fenton processes for degradation of antineoplastic agents in water matrices: Current knowledge and future challenges evaluation using a bibliometric and systematic analysis. Environ. Sci. Pollut. Res. Int..

[8] Gao L., Cao Y., Wang L., Li S. (2021). A review on sustainable reuse applications of Fenton sludge during wastewater treatment. Front. Environ. Sci. Eng..

[9] Sun S.-P., Zeng X., Li C., Lemley A.T. (2014). Enhanced heterogeneous and homogeneous Fenton-like degradation of carbamazepine by nano-Fe_3_O_4_/H_2_O_2_ with nitrilotriacetic acid. Chem. Eng. J..

[10] De Luca A., Dantas R.F., Esplugas S. (2015). Study of Fe(III)-NTA chelates stability for applicability in photo-Fenton at neutral pH. Appl. Catal. B Environ..

[11] Luo H., Zeng Y., He D., Pan X. (2021). Application of iron-based materials in heterogeneous advanced oxidation processes for wastewater treatment: A review. Chem. Eng. J..

[12] Nowack B. (2002). Environmental chemistry of aminopolycarboxylate chelating agents. Environ. Sci. Technol..

[13] Chen J., Meng X., Tian Y., Wang X., Zhu J., Zheng H., Wang L. (2020). Fabrication of a superhydrophilic PVDF-g-PAA@FeOOH ultrafiltration membrane with visible light photo-fenton self-cleaning performance. J. Membr. Sci..

[14] Lin J., Tian W., Guan Z., Zhang H., Duan X., Wang H., Sun H., Fang Y., Huang Y., Wang S. (2022). Functional Carbon Nitride Materials in Photo-Fenton-Like Catalysis for Environmental Remediation. Adv. Funct. Mater..

[15] Liu X., Zhou Y., Zhang J., Luo L., Yang Y., Huang H., Peng H., Tang L., Mu Y. (2018). Insight into electro-Fenton and photo-Fenton for the degradation of antibiotics: Mechanism study and research gaps. Chem. Eng. J..

[16] Giannakis S. (2018). Analogies and differences among bacterial and viral disinfection by the photo-Fenton process at neutral pH: A mini review. Environ. Sci. Pollut. Res. Int..

[17] Gutierrez-Mata A.G., Velazquez-Martínez S., Álvarez-Gallegos A., Ahmadi M., Hernández-Pérez J.A., Ghanbari F., Silva-Martínez S. (2017). Recent Overview of Solar Photocatalysis and Solar Photo-Fenton Processes for Wastewater Treatment. Int. J. Photoenergy.

[18] Shao D.D., Yang W.J., Xiao H.F., Wang Z.Y., Zhou C., Cao X.L., Sun S.P. (2020). Self-Cleaning Nanofiltration Membranes by Coordinated Regulation of Carbon Quantum Dots and Polydopamine. ACS Appl. Mater. Interfaces.

[19] Zhang S., Hedtke T., Zhu Q., Sun M., Weon S., Zhao Y., Stavitski E., Elimelech M., Kim J.H. (2021). Membrane-Confined Iron Oxychloride Nanocatalysts for Highly Efficient Heterogeneous Fenton Water Treatment. Environ. Sci. Technol..

[20] Babuponnusami A., Muthukumar K. (2014). A review on Fenton and improvements to the Fenton process for wastewater treatment. J. Environ. Chem. Eng..

[21] Silwana N., Calderon B., Ntwampe S.K.O., Fullana A. (2020). Heterogeneous Fenton Degradation of Patulin in Apple Juice Using Carbon-Encapsulated Nano Zero-Valent Iron (CE-nZVI). Foods.

[22] Sciscenko I., Arques A., Escudero-Onate C., Roccamante M., Ruiz-Delgado A., Miralles-Cuevas S., Malato S., Oller I. (2021). A Rational Analysis on Key Parameters Ruling Zerovalent Iron-Based Treatment Trains: Towards the Separation of Reductive from Oxidative Phases. Nanomaterials.

[23] Minella M., Sappa E., Hanna K., Barsotti F., Maurino V., Minero C., Vione D. (2016). Considerable Fenton and photo-Fenton reactivity of passivated zero-valent iron. RSC Adv..

[24] Lee S., Bayarkhuu B., Han Y., Kim H.-W., Jeong S., Boo C., Byun J. (2022). Multifunctional photo-Fenton-active membrane for solar-driven water purification. J. Membr. Sci..

[25] Feng Y., Huang X., Wu H., Li D., Sun Y. (2022). Self-cleaning ceramic membranes coated with low crystalline Fe_2_O_3_@CMWCNTs for highly efficient photo-Fenton removal of aromatic compounds. J. Environ. Chem. Eng..

[26] Yan C., Cheng Z., Wei J., Xu Q., Zhang X., Wei Z. (2022). Efficient degradation of antibiotics by photo-Fenton reactive ceramic membrane with high flux by a facile spraying method under visible LED light. J. Clean. Prod..

[27] Xie A., Cui J., Yang J., Chen Y., Dai J., Lang J., Li C., Yan Y. (2019). Photo-Fenton self-cleaning membranes with robust flux recovery for an efficient oil/water emulsion separation. J. Mater. Chem. A.

[28] Zhou Y., Xiao B., Liu S.-Q., Meng Z., Chen Z.-G., Zou C.-Y., Liu C.-B., Chen F., Zhou X. (2016). Photo-Fenton degradation of ammonia via a manganese–iron double-active component catalyst of graphene–manganese ferrite under visible light. Chem. Eng. J..

[29] Gao J., Ma S., Xu M., Yuan M., Li J., Xue J., Wang M. (2022). Photo-Fenton superwettable NiFe_2_O_4_/TA/PVDF composite membrane for organic pollutant degradation with successively oil-in-water separation. Chemosphere.

[30] Gao C., Chen S., Quan X., Yu H., Zhang Y. (2017). Enhanced Fenton-like catalysis by iron-based metal organic frameworks for degradation of organic pollutants. J. Catal..

[31] Gao S., Cen W., Li Q., Li J., Lu Y., Wang H., Wu Z. (2018). A mild one-step method for enhancing optical absorption of amine-functionalized metal-organic frameworks. Appl. Catal. B Environ..

[32] Yang Z., Xu X., Liang X., Lei C., Cui Y., Wu W., Yang Y., Zhang Z., Lei Z. (2017). Construction of heterostructured MIL-125/Ag/g-C3N4 nanocomposite as an efficient bifunctional visible light photocatalyst for the organic oxidation and reduction reactions. Appl. Catal. B Environ..

[33] He X., Fang H., Gosztola D.J., Jiang Z., Jena P., Wang W.N. (2019). Mechanistic Insight into Photocatalytic Pathways of MIL-100(Fe)/TiO_2_ Composites. ACS Appl. Mater. Interfaces.

[34] Ahmad M., Chen S., Ye F., Quan X., Afzal S., Yu H., Zhao X. (2019). Efficient photo-Fenton activity in mesoporous MIL-100(Fe) decorated with ZnO nanosphere for pollutants degradation. Appl. Catal. B Environ..

[35] Zhang Y., Zhou J., Chen X., Wang L., Cai W. (2019). Coupling of heterogeneous advanced oxidation processes and photocatalysis in efficient degradation of tetracycline hydrochloride by Fe-based MOFs: Synergistic effect and degradation pathway. Chem. Eng. J..

[36] Yue R., Raisi B., Rahmatinejad J., Ye Z., Barbeau B., Rahaman M.S. (2021). A photo-Fenton nanocomposite ultrafiltration membrane for enhanced dye removal with self-cleaning properties. J. Colloid Interface Sci..

[37] Yu X., Lin X., Feng W., Li W. (2019). Enhanced catalytic performance of a bio-templated TiO_2_ UV-Fenton system on the degradation of tetracycline. Appl. Surf. Sci..

[38] Date M.K., Yang L.H., Yang T.Y., Wang K.Y., Su T.Y., Wu D.C., Cheuh Y.L. (2020). Three-Dimensional CuO/TiO_2_ Hybrid Nanorod Arrays Prepared by Electrodeposition in AAO Membranes as an Excellent Fenton-Like Photocatalyst for Dye Degradation. Nanoscale Res. Lett..

[39] Deemter D., Coelho F.E.B., Oller I., Malato S., Amat A.M. (2022). Assessment of a Novel Photocatalytic TiO_2_-Zirconia Ultrafiltration Membrane and Combination with Solar Photo-Fenton Tertiary Treatment of Urban Wastewater. Catalysts.

[40] Tian Y., Yao S., Zhou L., Hu Y., Lei J., Wang L., Zhang J., Liu Y., Cui C. (2022). Efficient removal of antibiotic-resistant bacteria and intracellular antibiotic resistance genes by heterogeneous activation of peroxymonosulfate on hierarchical macro-mesoporous Co_3_O_4_-SiO_2_ with enhanced photogenerated charges. J. Hazard. Mater..

[41] Mokoba T., Li Z., Zhang T.C., Yuan S. (2022). Superwetting sea urchin-like BiOBr@Co_3_O_4_ nanowire clusters-coated copper mesh with efficient emulsion separation and photo-Fenton-like degradation of soluble dye. Appl. Surf. Sci..

[42] Lan H., Wang F., Lan M., An X., Liu H., Qu J. (2019). Hydrogen-Bond-Mediated Self-Assembly of Carbon-Nitride-Based Photo-Fenton-like Membranes for Wastewater Treatment. Environ. Sci. Technol..

[43] Yue R., Saifur Rahaman M. (2022). Hydrophilic and underwater superoleophobic porous graphitic carbon nitride (g-C_3_N_4_) membranes with photo-Fenton self-cleaning ability for efficient oil/water separation. J. Colloid Interface Sci..

[44] Aouadja F., Bouzerara F., Guvenc C.M., Demir M.M. (2022). Fabrication and properties of novel porous ceramic membrane supports from the (Sig) diatomite and alumina mixtures. Boletín Soc. Española Cerámica Vidr..

[45] Xie A., Cui J., Yang J., Chen Y., Lang J., Li C., Yan Y., Dai J. (2020). Photo-Fenton self-cleaning PVDF/NH_2_-MIL-88B(Fe) membranes towards highly-efficient oil/water emulsion separation. J. Membr. Sci..

[46] Bao X., Liu Q., Yang J., Wang F., Yu F., Yu J., Yang Y. (2022). Cascading in-situ generation of H_2_O_2_ and Fenton-like reaction in photocatalytic composite ultrafiltration membrane for high self-cleaning performance in wastewater treatment. J. Membr. Sci..

[47] Lv Y., Zhang C., He A., Yang S.J., Wu G.P., Darling S.B., Xu Z.K. (2017). Photocatalytic Nanofiltration Membranes with Self-Cleaning Property for Wastewater Treatment. Adv. Funct. Mater..

[48] González-Bahamón L.F., Mazille F., Benítez L.N., Pulgarín C. (2011). Photo-Fenton degradation of resorcinol mediated by catalysts based on iron species supported on polymers. J. Photochem. Photobiol. A Chem..

[49] He Z., Lyu Z., Gu Q., Zhang L., Wang J. (2019). Ceramic-based membranes for water and wastewater treatment. Colloids Surf. A Physicochem. Eng. Asp..

[50] Bortot Coelho F.E., Deemter D., Candelario V.M., Boffa V., Malato S., Magnacca G. (2021). Development of a photocatalytic zirconia-titania ultrafiltration membrane with anti-fouling and self-cleaning properties. J. Environ. Chem. Eng..

[51] Nazemidashtarjandi S., Mousavi S.A., Bastani D. (2017). Preparation and characterization of polycarbonate/thermoplastic polyurethane blend membranes for wastewater filtration. J. Water Process Eng..

[52] Zheng S., Chen H., Tong X., Wang Z., Crittenden J.C., Huang M. (2021). Integration of a Photo-Fenton Reaction and a Membrane Filtration using CS/PAN@FeOOH/g-C_3_N_4_ Electrospun Nanofibers: Synthesis, Characterization, Self-cleaning Performance and Mechanism. Appl. Catal. B Environ..

[53] Wang D., Yang J., Yang H., Zhao P., Shi Z. (2022). Fe-complex modified cellulose acetate composite membrane with excellent photo-Fenton catalytic activity. Carbohydr. Polym..

[54] Liu D., Yin J., Tang H., Wang H., Liu S., Huang T., Fang S., Zhu K., Xie Z. (2021). Fabrication of ZIF-67@PVDF ultrafiltration membrane with improved antifouling and separation performance for dye wastewater treatment via sulfate radical enhancement. Sep. Purif. Technol..

[55] Xu B., Wang X., Huang Y., Liu J., Wang D., Feng S., Huang X., Wang H. (2020). Electrospinning preparation of PAN/TiO_2_/PANI hybrid fiber membrane with highly selective adsorption and photocatalytic regeneration properties. Chem. Eng. J..

[56] Vinh-Thang H., Kaliaguine S. (2013). Predictive models for mixed-matrix membrane performance: A review. Chem. Rev..

[57] Zheng Y., Wang L., Zhao G., Long X., Hu J., Jiao F. (2022). Photo-Fenton Antifouling Membrane Based on Hydrophilized MIL-88A for Sustainable Treatment of Colored Emulsions. Ind. Eng. Chem. Res..

[58] Domenzain-Gonzalez J., Castro-Arellano J.J., Galicia-Luna L.A., Rodriguez-Cruz M., Hernandez-Lopez R.T., Lartundo-Rojas L. (2021). Photocatalytic membrane reactor based on Mexican Natural Zeolite: RB5 dye removal by photo-Fenton process. J. Environ. Chem. Eng..

[59] Molina R., Segura Y., Martínez F., Melero J.A. (2012). Immobilization of active and stable goethite coated-films by a dip-coating process and its application for photo-Fenton systems. Chem. Eng. J..

[60] Li Q., Kong H., Li P., Shao J., He Y. (2019). Photo-Fenton degradation of amoxicillin via magnetic TiO_2_-graphene oxide-Fe_3_O_4_ composite with a submerged magnetic separation membrane photocatalytic reactor (SMSMPR). J. Hazard. Mater..

[61] Wang Q., Wang P., Xu P., Hu L., Wang X., Qu J., Zhang G. (2021). Submerged membrane photocatalytic reactor for advanced treatment of p-nitrophenol wastewater through visible-light-driven photo-Fenton reactions. Sep. Purif. Technol..

[62] Wang M., Xu Z., Hou Y., Li P., Sun H., Niu Q.J. (2020). Photo-Fenton assisted self-cleaning hybrid ultrafiltration membranes with high-efficient flux recovery for wastewater remediation. Sep. Purif. Technol..

[63] Yang H., Zhu B., Zhu L., Zeng Z., Wang G., Xiong Z. (2021). Efficient Fenton-Like Catalysis Boosting the Antifouling Performance of the Heterostructured Membranes Fabricated via Vapor-Induced Phase Separation and In Situ Mineralization. ACS Appl. Mater. Interfaces.

[64] Bao Z., Chen D., Li N., Xu Q., Li H., He J., Lu J. (2020). Superamphiphilic and underwater superoleophobic membrane for oil/water emulsion separation and organic dye degradation. J. Membr. Sci..

[65] Zhang S., Jiang G., Gao S., Jin H., Zhu Y., Zhang F., Jin J. (2018). Cupric Phosphate Nanosheets-Wrapped Inorganic Membranes with Superhydrophilic and Outstanding Anticrude Oil-Fouling Property for Oil/Water Separation. ACS Nano.

[66] Wang K., He H., Wei B., Zhang T.C., Chang H., Li Y., Tian X., Fan Y., Liang Y., Yuan S. (2021). Multifunctional Switchable Nanocoated Membranes for Efficient Integrated Purification of Oil/Water Emulsions. ACS Appl. Mater. Interfaces.

[67] Zhang L., He Y., Luo P., Ma L., Li S., Nie Y., Zhong F., Wang Y., Chen L. (2022). Photocatalytic GO/M88A “interceptor plate” assembled nanofibrous membrane with photo-Fenton self-cleaning performance for oil/water emulsion separation. Chem. Eng. J..

[68] Howarter J.A., Youngblood J.P. (2009). Amphiphile grafted membranes for the separation of oil-in-water dispersions. J. Colloid Interface Sci..

[69] Werber J.R., Osuji C.O., Elimelech M. (2016). Materials for next-generation desalination and water purification membranes. Nat. Rev. Mater..

[70] Wang Z., Lin S. (2017). The impact of low-surface-energy functional groups on oil fouling resistance in membrane distillation. J. Membr. Sci..

[71] Ma Z., Shu G., Lu X. (2020). Preparation of an antifouling and easy cleaning membrane based on amphiphobic fluorine island structure and chemical cleaning responsiveness. J. Membr. Sci..

[72] Jing J., Liu Z.-J., Zhang X.-G., Ren L.-N., Wang H.-Y. (2023). Research progress of superwetting oil-water separation membrane. Surf. Technol..

[73] Li Q., Kong H., Jia R., Shao J., He Y. (2019). Enhanced catalytic degradation of amoxicillin with TiO_2_-Fe_3_O_4_ composites via a submerged magnetic separation membrane photocatalytic reactor (SMSMPR). RSC Adv..

[74] Xie A., Cui J., Yang J., Chen Y., Lang J., Li C., Yan Y., Dai J. (2020). Graphene oxide/Fe(III)-based metal-organic framework membrane for enhanced water purification based on synergistic separation and photo-Fenton processes. Appl. Catal. B Environ..

[75] Yue R., Chen T., Ye Z., Barbeau B., Rahaman M.S. (2021). A photo-Fenton graphene oxide membrane with improved perm-selectivity and self-cleaning ability for efficient dye removal under visible light irradiation. J. Water Process Eng..

[76] Wang Y., Yang T., Chen J., Wen S., Li D., Wang B., Zhang Q. (2022). Multifunctional ferrocene-based photo-Fenton membrane: An efficient integration of rejection and catalytic process. Sep. Purif. Technol..

[77] Piao H., Zhao J., Liu M., Zhang S., Huang Q., Liu Y., Xiao C. (2022). Ultra-low power light driven lycopodium-like nanofiber membrane reinforced by PET braid tube with robust pollutants removal and regeneration capacity based on photo-Fenton catalysis. Chem. Eng. J..

[78] Zheng Y., Zhang C., Wang L., Long X., Zhang J., Zuo Y., Jiao F. (2021). Tannic acid-based complex coating modified membranes with photo-Fenton self-cleaning property for sustainable oil-in-water emulsion separation. Sep. Purif. Technol..

[79] Xie A., Wu Y., Liu Y., Xue C., Ding G., Cheng G., Cui J., Pan J. (2022). Robust antifouling NH_2_-MIL-88B coated quartz fibrous membrane for efficient gravity-driven oil-water emulsion separation. J. Membr. Sci..

[80] Gao Y., Yan S., He Y., Fan Y., Zhang L., Ma J., Hou R., Chen L., Chen J. (2021). A photo-Fenton self-cleaning membrane based on NH2-MIL-88B (Fe) and graphene oxide to improve dye removal performance. J. Membr. Sci..

[81] Zhou L., Xiao G., He Y., Wu J., Shi H., Yin X., He T., Li Z., Chen J. (2022). Multi-functional composite membrane with strong photocatalysis to effectively separate emulsified-oil/dyes from complex oily sewage. Colloids Surf. A Physicochem. Eng. Asp..

[82] Moreira V.R., Lebron Y.A.R., Couto C.F., Maia A., Moravia W.G., Amaral M.C.S. (2022). Process development for textile wastewater treatment towards zero liquid discharge: Integrating membrane separation process and advanced oxidation techniques. Process Saf. Environ. Prot..

[83] Fan D., Ding L., Huang H., Chen M., Ren H. (2017). Fluidized-bed Fenton coupled with ceramic membrane separation for advanced treatment of flax wastewater. J. Hazard. Mater..

[84] Estrada-Arriaga E.B., Zepeda-Aviles J.A., García-Sánchez L. (2016). Post-treatment of real oil refinery effluent with high concentrations of phenols using photo-ferrioxalate and Fenton’s reactions with membrane process step. Chem. Eng. J..

[85] Ioannou L.A., Michael C., Vakondios N., Drosou K., Xekoukoulotakis N.P., Diamadopoulos E., Fatta-Kassinos D. (2013). Winery wastewater purification by reverse osmosis and oxidation of the concentrate by solar photo-Fenton. Sep. Purif. Technol..

[86] Aydiner C., Mert B.K., Dogan E.C., Yatmaz H.C., Dagli S., Aksu S., Tilki Y.M., Goren A.Y., Balci E. (2019). Novel hybrid treatments of textile wastewater by membrane oxidation reactor: Performance investigations, optimizations and efficiency comparisons. Sci. Total Environ..

[87] Gholami M., Abbasi Souraki B., Pendashteh A., Bagherian Marzouni M. (2017). Efficiency evaluation of the membrane/AOPs for paper mill wastewater treatment. Env. Technol..

[88] Calik C., Cifci D.I. (2022). Comparison of kinetics and costs of Fenton and photo-Fenton processes used for the treatment of a textile industry wastewater. J. Environ. Manag..

[89] Sun S., Yao H., Fu W., Hua L., Zhang G., Zhang W. (2018). Reactive Photo-Fenton ceramic membranes: Synthesis, characterization and antifouling performance. Water Res..

[90] Liu F., Yao H., Sun S., Tao W., Wei T., Sun P. (2020). Photo-Fenton activation mechanism and antifouling performance of an FeOCl-coated ceramic membrane. Chem. Eng. J..

[91] Dogan E.C., Kilicoglu O., Narci A.O., Mert B.K., Durna E., Akbacak U.A., Aydiner C. (2021). Fenton and photo-Fenton processes integrated with submerged ultrafiltration for the treatment of pulp and paper industry wastewater. J. Environ. Chem. Eng..

[92] Alias N.H., Jaafar J., Samitsu S., Matsuura T., Ismail A.F., Othman M.H.D., Rahman M.A., Othman N.H., Abdullah N., Paiman S.H. (2019). Photocatalytic nanofiber-coated alumina hollow fiber membranes for highly efficient oilfield produced water treatment. Chem. Eng. J..

[93] Gao J., Chen W., Shi H., Li Z., Jing L., Hou C., Wang J., Wang Y. (2022). Co_3_O_4_@Fe_3_O_4_/cellulose blend membranes for efficient degradation of perfluorooctanoic acid in the visible light-driven photo-Fenton system. Surf. Interfaces.

[94] Vilardi G., Di Palma L., Verdone N. (2018). On the critical use of zero valent iron nanoparticles and Fenton processes for the treatment of tannery wastewater. J. Water Process Eng..

[95] Giannakis S., Polo López M.I., Spuhler D., Sánchez Pérez J.A., Fernández Ibáñez P., Pulgarin C. (2016). Solar disinfection is an augmentable, in situ -generated photo-Fenton reaction—Part 1: A review of the mechanisms and the fundamental aspects of the process. Appl. Catal. B Environ..

[96] Rincón A.-G., Pulgarin C. (2006). Comparative evaluation of Fe^3+^ and TiO_2_ photoassisted processes in solar photocatalytic disinfection of water. Appl. Catal. B Environ..

[97] Barreca S., Velez Colmenares J.J., Pace A., Orecchio S., Pulgarin C. (2015). Escherichia coli inactivation by neutral solar heterogeneous photo-Fenton (HPF) over hybrid iron/montmorillonite/alginate beads. J. Environ. Chem. Eng..

[98] Ruales-Lonfat C., Barona J.F., Sienkiewicz A., Vélez J., Benítez L.N., Pulgarín C. (2016). Bacterial inactivation with iron citrate complex: A new source of dissolved iron in solar photo-Fenton process at near-neutral and alkaline pH. Appl. Catal. B Environ..

[99] Ortega-Gomez E., Esteban Garcia B., Ballesteros Martin M.M., Fernandez Ibanez P., Sanchez Perez J.A. (2014). Inactivation of natural enteric bacteria in real municipal wastewater by solar photo-Fenton at neutral pH. Water Res..

[100] Polo-López M.I., Oller I., Fernández-Ibáñez P. (2013). Benefits of photo-Fenton at low concentrations for solar disinfection of distilled water. A case study: Phytophthora capsici. Catal. Today.

[101] Ortega-Gomez E., Fernandez-Ibanez P., Ballesteros Martin M.M., Polo-Lopez M.I., Esteban Garcia B., Sanchez Perez J.A. (2012). Water disinfection using photo-Fenton: Effect of temperature on Enterococcus faecalis survival. Water Res..

[102] Aurioles-López V., Polo-López M.I., Fernández-Ibáñez P., López-Malo A., Bandala E.R. (2016). Effect of iron salt counter ion in dose–response curves for inactivation of Fusarium solani in water through solar driven Fenton-like processes. Phys. Chem. Earth Parts A/B/C.

[103] Giannakis S., Ruales-Lonfat C., Rtimi S., Thabet S., Cotton P., Pulgarin C. (2016). Castles fall from inside: Evidence for dominant internal photo-catalytic mechanisms during treatment of Saccharomyces cerevisiae by photo-Fenton at near-neutral pH. Appl. Catal. B Environ..

[104] Ruales-Lonfat C., Benítez N., Sienkiewicz A., Pulgarín C. (2014). Deleterious effect of homogeneous and heterogeneous near-neutral photo-Fenton system on Escherichia coli. Comparison with photo-catalytic action of TiO_2_ during cell envelope disruption. Appl. Catal. B Environ..

[105] Giannakis S., Voumard M., Rtimi S., Pulgarin C. (2018). Bacterial disinfection by the photo-Fenton process: Extracellular oxidation or intracellular photo-catalysis?. Appl. Catal. B Environ..

[106] Xue J., Yuan M., Gao J., Zhang Z., Wang M., Ma S. (2023). Photo-Fenton catalyst Fe(III)@PCN-222 grafted on PVDF membrane for multitasking applications: Oil/water separation, aromatic pollutants degradation and bacterial inactivation. Process Saf. Environ. Prot..

[107] Ballesteros Martin M.M., Garrido L., Casas Lopez J.L., Sanchez O., Mas J., Maldonado M.I., Sanchez Perez J.A. (2011). An analysis of the bacterial community in a membrane bioreactor fed with photo-Fenton pre-treated toxic water. J. Ind. Microbiol. Biotechnol..

[108] Sakurai K.S.I., Neves L.C., Souza J.B.D., Vidal C.M.D.S., Souza K.V.D. (2016). Pós-tratamento de efluente de indústria de papel e celulose empregando membranas de microfiltração e ultrafiltração combinadas com o processo foto-fenton. Sci. For..

[109] Yousif E., Haddad R. (2013). Photodegradation and photostabilization of polymers, especially polystyrene: Review. Springerplus.

[110] Lee M.J., Ong C.S., Lau W.J., Ng B.C., Ismail A.F., Lai S.O. (2016). Degradation of PVDF-based composite membrane and its impacts on membrane intrinsic and separation properties. J. Polym. Eng..

